# Impact du COVID-19 sur la consultation en ophtalmologie en Maroc: enquête auprès de 35 ophtalmologistes

**DOI:** 10.11604/pamj.2020.36.163.23468

**Published:** 2020-07-08

**Authors:** Shamil Louaya, Omar Moustaine, Mohammed badaoui, Youssef Hnach, Ahmed Alaayoud, Said Chatoui

**Affiliations:** 1 Service d´ophtalmologie, 1 CMC, Maroc,; 2 Service d´ophtalmologie, CHU d´Agadir, Maroc,; 3 Service de médecine interne, Maroc,; 4 Service de gastro-entérologie, 1 CMC, Maroc,; 5 Service de néphrologie, 1 CMC, Faculté de médecine et de pharmacie d´Agadir, Université Ibn Zohr, Agadir, Maroc

**Keywords:** Covid 19, consultation ophtalmologique, confinement, COVID-19, ophthalmology consultation, containment

## Abstract

D´une épidémie en Décembre à une pandémie mondiale en début de Mars, le COVID-19 est arrivée au Maroc le 02 Mars et le nombre des patients consultants en ophtalmologie a connu, depuis, une baisse considérable. Par ailleurs, les ophtalmologistes en majorité se sont portés au-devant de la scène pour assurer la continuité de soins et les urgences en respectant les consignes sanitaires. Nous avons élaboré un questionnaire afin de collecter les informations sur les orientations générales de 35 ophtalmologistes concernant l´impact du COVID-19 sur l´activité de la consultation. Les résultats de cette enquête ont objectivé que 88,57% des ophtalmologistes sondés ont maintenu leurs activités de consultation dont les ¾ faisaient que les cas urgents ou dont l´état nécessite une prise en charge sans différée. La majorité des ophtalmologistes déclare une activité en baisse d´au moins 90% par rapport à leur activité habituelle. Les ophtalmologues en activité estiment que le risque d´être contaminer ou de contaminer ses patients et ses proches demeures moyennes à élever dans la majorité des cas malgré les gestes barrières de protection.

## Abstract (en)

From an epidemic in December to a global pandemic in early March, COVID-19 arrived on Moroccan soil on March 2nd and ophthalmology consultations decreased considerably. The majority of ophthalmologists come to the fore to ensure continuity of care and emergency care following health regulations. We developed a questionnaire to collect information on the general approach of 35 ophthalmologists regarding the impact of COVID -19 on consultation activities. The results of the survey objectified that 88.57% of the ophthalmologists surveyed maintained their consultation activities; ¾ of them only treated urgent cases or patients whose condition required undelayed management. The majority of ophthalmologists reported a decrease in consultations of at least 90% compared to their standard workflow. Active ophthalmologists believe that the risk of being infected or infecting their patients and others ranges from medium to high in the majority of cases despite protective barrier gestures.

## Introduction

Au décours de cette pandémie du COVID-19, les ophtalmologistes marocains sont fortement mobilisés, depuis le début, pour répondre aux besoins de prise en charge des patients. Que ce soit des affections ophtalmologiques ayant un caractère urgent ou non, certains soins et examens sont continuellement maintenus, tant à l'hôpital public qu´au sein des cabinets privés. À ce titre, une organisation spontanée et collaborative a été instaurée, ayant pour objectif d´assurer les soins de base nécessaires et visant un meilleur suivi des pathologies chroniques. Un questionnaire a été élaboré afin de collecter les informations sur les orientations générales de 35 ophtalmologistes concernant l´impact du COVID-19 sur l´activité de la consultation.

## Méthodes

Un questionnaire a été élaboré sur la plateforme Google-Forms portant sur 35 ophtalmologistes. L'ensemble des données collectées a été saisi sur le logiciel IBM SPSS version 20. Dans un premier temps nous avons procédé à une analyse descriptive des caractéristiques étudiées. Les résultats ont été présentés sous forme de pourcentage pour les variables qualitatives. Ensuite une analyse comparative a été faite par le test d'indépendance khi deux. La signification statistique a été définie par p (degré de signification <0,05).

## Résultats

**Figure 1:**
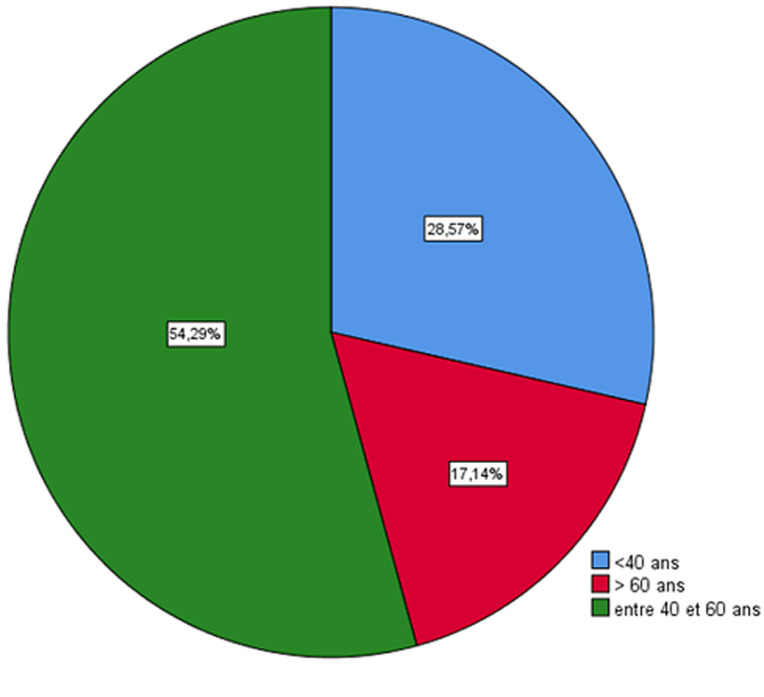
répartition selon le sexe

**Figure 2:**
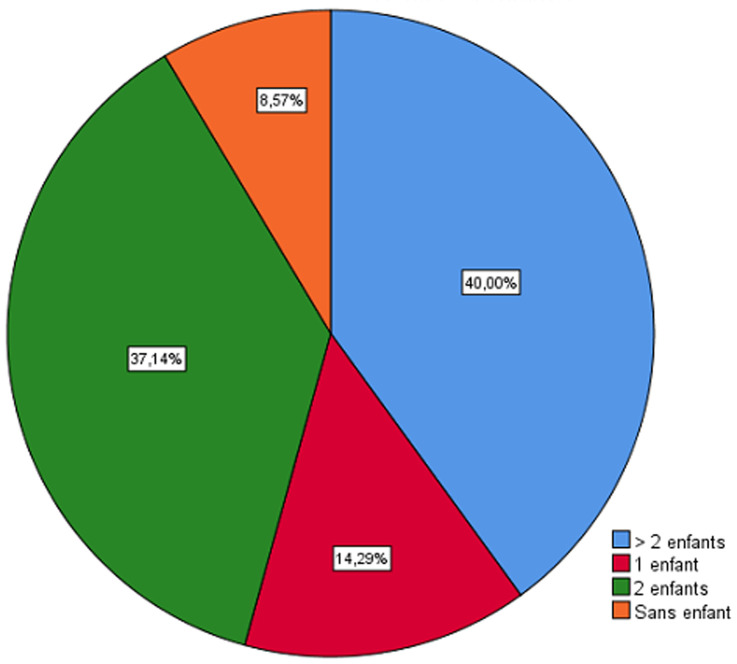
répartition selon le nombre d´enfants

Les caractéristiques démographiques des ophtalmologistes participants à cette étude ont objectivé un sex-ratio H/F de 3,3 ; environ 30% sont âgés de plus de 60 ans (Figure 1), plus de 90% ont un enfant ou plus (Figure 2). Par ailleurs environ 90% ont maintenu la consultation médicale au secteur libéral contre 100% au secteur public. Le tiers des 90% ont prodigué une consultation ordinaire (cas urgent ou non). Seulement 3% ont maintenu une activité opératoire en dehors des urgences. Les mesures barrières sont globalement adoptées à l´unanimité, ainsi 100% utilisaient fréquemment les solutions hydro-alcooliques, 97% des masque de protection respiratoire avec 35% des masques FFP2 et 65% des masques chirurgicaux, les surblouses à usage unique dans 23%, les gants ainsi que les charlottes sont utilisés dans plus 60% des cas. Dans le questionnaire on a demandé aux ophtalmologistes d´évaluer le risque d´être contaminé par le COVID-19 pendant la consultation ophtalmologique et 45,7% ont opté pour un risque moyen (surtout chez ceux qui ont maintenus une consultation normale avec (p =0,022)) contre 37,1% pour un risque élevé surtout pour les participantes d´une façon significative (p =0,034) et le reste (soit 17,2%) pour un risque faible. D´autre part et concernant le risque de contaminer les patients ou les proches, le tiers des participants l´ont évalué à élever, le tiers à moyen et le tiers restant à faible. Les participants ayant maintenus une consultation normale estimaient le risque de contaminer les patients ou les proches plus élevé que ceux qui ne font que des consultations urgentes (p=0,004).

## Discussion

Durant cette période de pandémie, La baisse du nombre des consultants a été bien remarquée auprès de tous les ophtalmologues, du fait de confinement obligatoire ainsi que de la peur d´être contaminé (85%). La majeure partie des patients, qui consultent pour problème de correction optique sans signe alarmant, renonce à la consultation et demande le report par téléphone. Les mesures barrières en ophtalmologie touchent à la fois tous les actants de la consultation (patient, secrétariat, salle d´attente, Unité de consultation et examens complémentaires, l´ophtalmologue) ainsi des recommandations à destination des ophtalmologistes, qui soulignent la rareté des conjonctivites en cas d'infection COVID-19 (1% environ), mais rappellent la nécessaire prudence et les mesures de précaution à adopter lors de l'examen d'un patient ayant une conjonctivite [1]. La majorité des infections par COVID-19 est bénigne (80%), voire asymptomatique. Les personnes à risque de complications parfois mortelles sont les personnes âgées et les personnes atteintes de pathologies chroniques. Selon Académie Française d´Ophtalmologie, La contamination ophtalmologiste - patient ou vice versa, se fait par contact rapproché par projection de gouttelettes naso-pharyngées ou par contact par des mains souillées (par patient et environnement), qui vont apporter le virus par impaction sur les muqueuses du visage, essentiellement du nez et de la bouche, et peut être sur les conjonctives. La transmission par aérosol (petites gouttelettes) est possible mais parait rare, sur la base des connaissances pour les autres coronavirus (SRAS et MERS), surtout à l´occasion de manœuvres respiratoires invasives [1].

La contamination peut aussi être manuportée si le soignant met la main sur son visage après contact d´une surface contaminée, sachant que le virus peut rester sur les surfaces jusqu´à 6h. L'ophtalmologiste est à risque accru du fait de sa proximité de la bouche et du nez des patients lors de l´examen à la lampe à fente, puisqu´un des facteurs de risque validé est la tenue « à distance de moins d´un mètre pendant plus de 15 minutes ». Par ailleurs, des conjonctivites peuvent faire partie de la maladie COVID même si elles semblent rares, aux alentours de 1%. Une excrétion virale dans les larmes a été trouvée seulement chez les patients ayant une conjonctivite. Dans ce contexte, il est prudent de prendre des précautions pour examiner les patients ayant une conjonctivite. Selon plusieurs études [2-5], les conjonctives et les larmes pourraient être une voie de transmission du SARS-CoV-2. Cependant, cette possibilité apparaît rare. Quant à aux signes ophtalmologiques pouvant être attribués à la COVID-19, ils sont, d'une façon générale, peu fréquents. Au cours des consultations ophtalmologiques et particulièrement lors de l´examen en lampe à fente, la distance rapprochée entre le médecin et le patient ainsi que le contact de l´ophtalmologiste avec les larmes du patient peuvent constituer une situation à risque aussi bien pour l´un que pour l´autre [6] des mesures barrières sont pratiquées par plus de 60% des ophtalmologistes participants dans notre étude en utilisant des gants, des surblouses et des papiers transparents de protection sur la lampe à fente.

D´après une enquête menée par le SNOF [7] auprès de 1500 ophtalmologistes en France, 60% des cabinets sont toujours ouverts contre 8,57% dans notre série, mais les patients s´y font rares. 82% des ophtalmologistes déclarent une activité en baisse d´au moins 95% par rapport à leur activité habituelle même si 75% assurent physiquement la prise en charge des demandes urgentes, et les deux tiers maintiennent des soins aux patients nécessitant un suivi ne pouvant être reporté, L´amplitude horaire d´ouverture des cabinets est inférieure à 10h par semaine pour plus de 3 cabinets sur 4 (78%). Dans un peu plus de la moitié des cas (53,5%), le cabinet est ouvert en présence d´une secrétaire. La baisse d´activité de consultation s´explique par plusieurs facteurs, selon les ophtalmologistes, parmi lesquels on cite : les consignes de sortir le moins possible, de limiter les déplacements au maximum, de reporter à plus tard tout ce qui n´est pas indispensable (91,7%) et enfin la peur des patients d´attraper le virus en venant en consultation sont pointés de doigt comme étant les facteurs principaux de leur baisse d´activité [8]. Par ailleurs, les ophtalmologistes attribuent aussi cette baisse d´activité à la crainte des patients d´être verbalisé en cas de contrôle par les forces de l´ordre (13,5%), ou encore à la réduction des moyens de transport (10%).

## Conclusion

Au cours de cette pandémie de COVID-19, le nombre des patients consultants en ophtalmologie a connu une baisse considérable. Les ophtalmologistes en majorité se sont portés au-devant de la scène pour assurer la continuité de soins et les urgences en respectant les consignes sanitaires. Les résultats de notre enquête portant sur 35 ophtalmologistes et concernant l´impact du COVID-19 sur l´activité de la consultation ont objectivé que 88,57% des ophtalmologistes sondés ont maintenu leurs activités de consultation dont les ¾ faisaient que les cas urgents. La majorité des ophtalmologistes déclare une activité en baisse d´au moins 90% par rapport à leur activité habituelle. Les ophtalmologues en activité estiment que le risque d´être contaminer ou de contaminer ses patients et ses proches demeure moyen à élevé dans la majorité des cas malgré les gestes barrières de protection.

### Etat des connaissances sur le sujet

- La pandémie de COVID-19 à beaucoup retenti sur l’activité de la consultation médicale;
- Plusieurs médecins ont différé les consultations non urgentes;
- Une réorganisation totale des sites de consultation a été entreprise chez tous les ophtalmologues avec adoption des mesures barrières sur tous les niveaux.

### Contribution de notre étude à la connaissance

- Cette étude montre l’impact de la pandémie COVID-19 sur l’activité de 35 ophtalmologues;
- Les ophtalmologues ont su s’adapter très vite et efficacement avec la pandémie pour ne pas laisser les patients graves ou urgents souffrir de leurs maladies;
- Les patients ayant des pathologies chroniques ophtalmologiques ont subi un retentissement négatif indirect.
